# Psychometric benefits of self-chosen rating scales over given rating scales

**DOI:** 10.3758/s13428-024-02429-w

**Published:** 2024-05-06

**Authors:** Tanja Kutscher, Michael Eid

**Affiliations:** 1 Leibniz Institute for Educational Trajectories, Department Research Data Center, Methods Development | Scaling and Test Design, Wilhelmsplatz 3, 96047 Bamberg, Germany; 2 Department of Psychology, Division of Methods and Evaluation, Freie Universitaet Berlin, Habelschwerdter Allee 45, 14195 Berlin, Germany

**Keywords:** Flourishing scale, Self-chosen rating scale, Inappropriate category use, Response styles, Mixture distribution IRT approach

## Abstract

Rating scales are susceptible to response styles that undermine the scale quality. Optimizing a rating scale can tailor it to individuals’ cognitive abilities, thereby preventing the occurrence of response styles related to a suboptimal response format. However, the discrimination ability of individuals in a sample may vary, suggesting that different rating scales may be appropriate for different individuals. This study aims to examine (1) whether response styles can be avoided when individuals are allowed to choose a rating scale and (2) whether the psychometric properties of self-chosen rating scales improve compared to given rating scales. To address these objectives, data from the flourishing scale were used as an illustrative example. MTurk workers from Amazon’s Mechanical Turk platform (*N* = 7042) completed an eight-item flourishing scale twice: (1) using a randomly assigned four-, six-, or 11-point rating scale, and (2) using a self-chosen rating scale. Applying the restrictive mixed generalized partial credit model (rmGPCM) allowed examination of category use across the conditions. Correlations with external variables were calculated to assess the effects of the rating scales on criterion validity. The results revealed consistent use of self-chosen rating scales, with approximately equal proportions of the three response styles. Ordinary response behavior was observed in 55–58% of individuals, which was an increase of 12–15% compared to assigned rating scales. The self-chosen rating scales also exhibited superior psychometric properties. The implications of these findings are discussed.

## Introduction

Rating scales are extensively employed in social and behavioral research to assess personality traits and attitudes. These scales allow individuals to express their degree of agreement with scale items by selecting a response category from a predefined set of ordered response options. The widespread use of rating scales can be attributed to their suitability for addressing various types of questions, as well as their ease of use. However, a substantial drawback of rating scales is their susceptibility to inappropriate category use (ICU; Wetzel et al., 2016a, 2016b; Van Vaerenbergh & Thomas, 2013). ICU arises when participants deviate from the intended use of the response categories and leads to systematic measurement error.

Recent research has provided several approaches to addressing ICU in data collected with rating scales. One approach aims to identify an optimal rating scale that minimizes ICU (e.g., Chen et al., 2015; Kutscher & Eid, 2020; Moors et al., 2014; Weijters et al., 2010a, 2010b, 2010c). The goal of this approach is to optimize the properties of rating scales (e.g., the number of response options, labels of response categories) to increase their robustness to ICU. Another area of research has focused on developing model-based approaches to address response styles (RSs), which are viewed as a form of ICU (for an overview, refer to Henninger & Meiser, 2020a, 2020b). These model-based approaches can be broadly classified into two categories: exploratory approaches and ad hoc approaches. Exploratory approaches identify the heterogeneity in scale usage during data analysis, allowing post hoc interpretation of RSs. These approaches include, for example, mixture distribution item response theory (IRT) models (for an overview, see von Davier & Carstensen, 2007) and multidimensional IRT models with random item-specific parameter estimates as additional dimensions (e.g., Adams et al., 2019; Henninger, 2021; Wang & Wu, 2011). In contrast, ad hoc approaches require researchers to make assumptions about the presence of specific RSs in the data before modeling and subsequently incorporate these RSs as additional dimensions or factors in the model. One example of this type of approach is the multidimensional nominal response model for estimating multiple latent traits and RS dimensions proposed by Falk and Cai (2016). Ad hoc models also include IRTree models, which assume that observed response behavior results from pre-defined multiple response processes (e.g., Böckenholt, 2012; De Boeck & Partchev, 2012). Both exploratory and ad hoc models provide individual trait value estimates that are adjusted for RS effects. Another, more contemporary trend in research involves gathering data using alternative response formats that are less prone to ICU, including the forced-choice response format (e.g., Wetzel et al., 2021).

However, these approaches possess certain limitations. First, determining a universal rating scale that is optimal for all constructs and samples is challenging, as the optimal features of a rating scale may differ based on the complexity of the construct or the attributes of the sample (Kieruj & Moors, 2013). For instance, student samples may appropriately employ rating scales with more response categories compared to the general population (Lozano et al., 2008; Weijters et al., 2010a, 2010b, 2010c). Furthermore, interindividual heterogeneity within a sample, including differences in individuals’ discrimination ability, cognitive capacity, education level, and experience, can result in the manifestation of RSs for certain individuals, even when apparently optimal rating scales are employed (Jin & Wang, 2014). Thus, establishing a one-size-fits-all rating scale is difficult. Second, utilizing mixed IRT models as a post hoc method to control for RS effects requires large sample sizes to estimate the model parameters accurately (Kutscher et al., 2019; Sen & Cohen, 2023). Conversely, ad hoc approaches rely on concrete assumptions about the presence of RSs in the data (e.g., Falk & Cai, 2016; Lyu & Bolt, 2022; Tutz et al., 2018; Wetzel & Carstensen, 2017). However, the prevalence of RSs can differ across constructs, and diverse populations may exhibit different RSs (cf. Carter et al., 2011; Eid & Rauber, 2000; Kieruj & Moors, 2013; Kim & Bolt, 2021). For example, when measuring job satisfaction in a representative sample of Australian employees, three RSs were identified: the extreme response style (ERS), characterized by a preference for extreme categories; the semi-ERS, displaying a preference for two extreme categories at each end of the response format; and the ordinary response style, which implies that each response category has at least one segment on the latent continuum in which it exhibits the highest probability of response, making it more likely to be endorsed than any other response category (ORS; Kutscher et al., 2017). In contrast to the Australian employees, for the same construct and using the same statistical method, a different set of response styles was observed in a sample of U.S. MTurk workers, who potentially possess more experience with responding to questionnaires. Specifically, the presence of the ERS, non-ERS (a tendency to avoid extreme categories), and ORS was found (Kutscher & Eid, 2020). Since the ERS appears to be a dominant response style (see e.g., Bolt & Johnson, 2009; Henninger, 2021), it is likely to be present in many datasets. Hence, ad hoc approaches fail to account for RS effects if the actual RSs present in the data are not included in the model (Schoenmakers et al., 2023). Furthermore, an approach’s effectiveness in eliminating RS effects can vary for the same dataset (see e.g., Scharl & Gnambs, 2022; Schoenmakers et al., 2023; Ulitzsch et al., 2023; Wetzel et al., 2016a, 2016b). Third, unlike rating scales, alternative response formats are challenging to implement (Dykema et al., 2022; Harzing et al., 2009; Wetzel & Frick, 2020), as these formats are more time-consuming and demanding to respond to (Sass et al., 2020; Wetzel et al., 2020). They are also generally less favored by respondents (Koskey et al., 2013). Moreover, alternative response formats do not necessarily outperform rating scales in terms of psychometric data quality (e.g., Koskey et al., 2013; Kreitchmann et al., 2019; Sung & Wu, 2018; Wetzel & Frick, 2020; Zhang et al., 2023), and they may not prevent the occurrence of response styles that are induced by stable person dispositions (e.g., Bäckström & Björklund, 2023; Böckenholt, 2017; Henninger et al., 2022; Liu et al., 2015; Wetzel et al., 2021).

Given the limitations of these approaches, employing self-chosen rating scales to prevent RSs may be promising. First, this approach assumes that individuals choose a rating scale that best matches their preferences. As a result, they possess a better understanding of the scale and the meaning of the response categories, enabling them to use the chosen rating scale appropriately. Second, previous research has demonstrated that self-defined response formats (e.g., self-defined labels for extreme categories or self-defined number of response categories) can assess constructs as effectively as traditional rating scales. Specifically, self-defined rating scales produce data with comparable or even superior levels of reliability and validity compared to traditional rating scales (Chami-Castaldi, 2012; Hofmans et al., 2009; Hofmans et al., 2007; Hofmans & Theuns, 2010). In addition, the utilization of self-defined rating scales may lead to higher data quality because participants are more involved in the response process and more accurately reflect their thoughts (Chami-Castaldi, 2012). Consequently, self-chosen rating scales are expected to be robust to trait-unrelated ICU, as they are tailored to individuals’ cognitive processes. Hence, this study offered respondents a variety of rating scales and encouraged them to choose the one that most closely suited their preferences. In doing so, the study investigated the occurrence of ICU and examined the psychometric properties of both self-chosen and given rating scales, comparing their effectiveness.

### Inappropriate category use as a source of bias for validity and reliability

Response style (RS), as a form of ICU, denotes a systematic tendency to respond to questionnaire items based on factors that are unrelated to the item’s specific content (Paulhus, 1991, p. 17). For instance, two participants who have chosen identical response categories may actually differ in their underlying trait levels, as one participant may have chosen the response category due to his or her individual response style, whereas the other participant may have used the rating scale in the intended manner. In the former case, the response becomes confounded with the RS (for more details, see Wetzel et al., 2016a, 2016b; Van Vaerenbergh & Thomas, 2013). As a result, the trait or attitude is not adequately measured by the questionnaire item (Baumgartner & Steenkamp, 2001; Podsakoff et al., 2003; Savalei & Falk, 2014). Thus, the presence of RSs in the data impairs construct validity. Recent research has provided evidence that RSs are prevalent in a substantial portion of samples (11–60%; Carter et al., 2011; Kutscher et al., 2017; Kutscher & Eid, 2020; Meiser & Machunsky, 2008; Wetzel et al., 2013), and the extent of variance attributable to RSs can fluctuate up to 43% (Tempelaar et al., 2020). This presence of RSs can bias estimates of means and variances (Schoenmakers et al., 2023; Weijters et al., 2010b), affect the shape and location of response distributions (Cheung & Rensvold, 2000; Mõttus et al., 2012; Reynolds & Smith, 2010), and impact the dimensional structure of a trait or attitude (Aichholzer, 2014; Jin & Wang, 2014; Navarro-González et al., 2016). Furthermore, the presence of RSs in the data can jeopardize the criterion validity of a scale of interest, leading to inflated or deflated correlation and regression coefficients, as well as biased estimates of model parameters (Khorramdel & von Davier, 2014; Moors, 2012; Morren et al., 2012; Plieninger, 2017; Rossi et al., 2001; Tutz et al., 2018; Weijters et al., 2008). In the context of multi-group analyses, RSs can also present a significant threat to measurement invariance (Liu et al., 2017). Thus, failure to account for the effects of RSs threatens the validity of conclusions drawn from analyses (Eid & Rauber, 2000). However, the impact of RSs is typically negligible when they are weakly correlated or uncorrelated with the latent trait (Plieninger, 2017; Wetzel et al., 2016a, 2016b) or when the influence of RSs is counteracted by the design of the scale. For example, the effect of the acquiescent response style (ARS), characterized as a tendency to agree with the item regardless of its content, can be neutralized on balanced scales with an equal number of positively and negatively worded items (Ferrando & Lorenzo-Seva, 2010; Primi et al., 2020).

Another form of ICU is so-called shortcut strategies, such as ignoring superfluous response categories, preferring labeled categories, choosing response categories in a particular area of a rating scale, or exhibiting a narrow response range (Andrich, 2010; Baumgartner & Steenkamp, 2001; Krosnick, 1999; Van Vaerenbergh & Thomas, 2013; Viswanathan et al., 2004; Wetzel & Carstensen, 2014). Shortcut strategies can coincide with RSs. For instance, respondents using the ERS also tend to ignore unnecessary response categories, reducing the rating scale to a few subjectively meaningful categories (Eid & Rauber, 2000; Meiser & Machunsky, 2008; Kutscher & Eid, 2020; Kutscher et al., 2017; Wu & Huang, 2010). This mismatch between predetermined and subjectively perceived response categories indicates that the predetermined response format does not adequately represent the underlying continuous trait, thereby violating the assumptions of the rating scale (Meiser & Machunsky, 2008) and impairing its reliability (De Jong et al., 2008; Dolnicar & Grün, 2009; Jin & Wang, 2014; Plieninger, 2017; Weijters et al., 2008).

Indeed, two components of ICU can be identified. The first refers to the ICU resulting from the response format (Cabooter et al., 2017). This component occurs when respondents have difficulties in understanding the meaning of the response categories, which can result from an inadequately designed rating scale (Krosnick, 1999). The other component includes ICU caused by individual dispositions (e.g., general self-efficacy; Kieruj & Moors, 2010, 2013; Kutscher & Eid, 2020; Moors, 2008; Moors et al., 2014). This component remains stable across different content areas (e.g., Weijters et al., 2010a; Wetzel et al., 2013) and shows consistency over several years (e.g., Aichholzer, 2013; Weijters et al., 2010b). Thus, ICU due to individual dispositions can be expected even with an optimal or individually tailored rating scale. This study focused on ICU due to the response format. We expect that when self-chosen rating scales are used, respondents will be better able to handle them, resulting in less ICU and data with higher psychometric quality. Therefore, we aim to examine the extent to which self-chosen rating scales can mitigate ICU due to response format and improve the psychometric quality of data.

### Rating scales and inappropriate category use

Inappropriate category use arising from a suboptimal rating scale can be prevented by offering an optimal rating scale that effectively conveys respondents’ judgments about item content without overburdening their cognitive capacities (Cox, 1980; Greenleaf, 1992; Krosnick, 1999; Lozano et al., 2008; Viswanathan et al., 2004; Weijters et al., 2008). According to the response process model proposed by Tourangeau and colleagues (Tourangeau et al., 2000), cognitive overload may occur when respondents encounter difficulty choosing a suitable response category from a given response format. This overload occurs when the rating scale does not account for the complexity of respondents’ thought processes regarding the item content (e.g., black-and-white thinking or sophisticated thinking) or their ability to discriminate between response categories (Baumgartner & Steenkamp, 2001; Cox, 1980; Krosnick, 1991; Naemi et al., 2009; Viswanathan et al., 2004). Suboptimal rating scales can lead to cognitive overload, resulting in ICU rather than ordinary responses (Arce-Ferrer, 2006; Baumgartner & Steenkamp, 2001; Cox, 1980; Greenleaf, 1992; Hamby & Levine, 2016; Krosnick, 1991; Swait & Adamowicz, 2001; Viswanathan et al., 2004; Weathers et al., 2005).

Past studies have supported this perspective, consistently providing empirical evidence for ICU’s occurrence when manipulating diverse features of rating scales, such as the number of response categories (Hamby & Levine, 2016; Kieruj & Moors, 2010, 2013; Moors, 2008; Weijters et al., 2010a, 2010b, 2010c; Harzing et al., 2009), labeling of response categories (fully labeled or labeled endpoints; Lau, 2007; Moors et al., 2014; Weijters et al., 2010a, 2010b, 2010c), numbering of response categories (increasing positive values, decreasing positive values, negative and positive values, or no numbering; Cabooter et al., 2016; Chyung et al., 2018; Gummer & Kunz, 2021; Moors et al., 2014; Schwarz et al., 1991), scale direction (a positive/high adjective on the left side/top of the scale and a negative/low adjective on the right side/bottom of the scale or in the reverse order; Liu & Keusch, 2017), and scale format (horizontal format, vertical format; Weijters et al., 2021). Regarding the number of response categories, recent research has suggested that the use of shorter rating scales with four to six response categories is more appropriate for most researchers’ purposes (Freund et al., 2013; Khadka et al., 2012; Kutscher & Eid, 2020). Shorter rating scales generally outperform longer ones in sufficiently capturing the full range of the underlying trait and maintaining the correct order of equidistant categories (Khadka et al., 2012; Kutscher & Eid, 2020). In contrast, longer rating scales may evoke disordered categories, potentially undermining the representation of the continuity of the underlying trait (Meiser & Machunsky, 2008). Furthermore, research investigating the impact of rating scale lengths on the reliability and validity of the scale has cautioned against employing rating scales with fewer than four or more than six or seven response options (Alwin et al., 2018; Culpepper, 2013; Eutsler & Lang, 2015; Lee & Paek, 2014; Lozano et al., 2008; Maydeu-Olivares et al., 2009; Müssig et al., 2022; Simms et al., 2019; for review, see DeCastellarnau, 2018; Taherdoost, 2019). This is because most individuals have limited ability to accurately discriminate between more than six or seven different units (Miller, 1956).

However, collecting data using an apparently optimal rating scale is only a partial solution, as identifying a universally optimal rating scale for all respondents within a particular population is problematic. Factors such as educational background, attitudes towards cognitive challenges, and individual preferences may determine which type of rating scale is appropriate for individuals with certain characteristics (e.g., Chami-Castaldi, 2012). Given the heterogeneity of individuals within a particular population, different rating scales may be optimal for different individuals. Thus, allowing individuals to respond to items using personally selected rating scales appears to be a potential solution for eliminating trait-unrelated ICU. The present study aims to investigate whether self-chosen rating scales lead to reduced ICU-related bias and yield improved psychometric properties compared to given rating scales.

### The present paper

The primary objective of the present study is to compare the psychometric properties of a given construct under two conditions: when participants provided responses using a self-chosen rating scale (self-chosen condition) and when they used a given rating scale (given condition). To achieve this objective, we used the flourishing scale, representing a key component of psychological well-being, as an illustrative example. Specifically, we investigated how inappropriate category use (ICU, including response styles) affects construct validity, criterion validity, and reliability. In both the self-chosen and given conditions, we employed three rating scales with four, six, and eleven categories. These rating scales were chosen on the basis that (i) an 11-point rating scale is commonly used to assess cognitive aspects of well-being (e.g., life satisfaction), and (ii) four- and six-point rating scales have been shown to be optimal for assessing similar constructs (Kutscher & Eid, 2020) and personality traits (Simms et al., 2019). Moreover, a six-point rating scale has been broadly endorsed in the research as the most suitable option due to its precision and user-friendly nature (e.g., Taherdoost, 2019). The two shorter rating scales do not include a middle category because individuals often misuse this category by refusing to provide any responses (Kulas & Stachowski, 2013; Lyu & Bolt, 2022; Murray et al., 2016; Nadler et al., 2015). Additionally, empirical findings have shown that even-numbered rating scales possess similar levels of reliability and validity as their odd-numbered counterparts (Alwin et al., 2018; Donnellan & Rakhshan, 2023; Simms et al., 2019). In both the self-chosen and given conditions, we applied the mixture distribution IRT approach to identify patterns of scale usage evoked by the rating scales (for an overview of finite mixture IRT models, see von Davier & Carstensen, 2007). This typological approach has the advantage of not requiring specific knowledge of additional variables that influence the response process or the imposition of distributional assumptions, for example regarding threshold parameters. The approach also enables the identification of discrete latent subpopulations that differ in their specific scale usage, even small ones, allowing for the estimation of the proportion of each latent subpopulation as a model parameter. Previous research has successfully used this approach to detect various types of ICU, including RSs, unsystematic response tendencies, faking, socially desirable responses, and avoidance of unnecessary response categories (e.g., Kutscher et al., 2020; Wetzel et al., 2013; Ziegler & Kemper, 2013). In addition, the mixture distribution IRT approach is suitable for analyzing data collected using short scales and a large number of response categories, as shown by Kutscher and colleagues’ (Kutscher et al., 2019) simulation study. We opted against using alternative psychometric modeling approaches, such as multidimensional IRT models with exploratory dimensions of response processes (as proposed by Adams et al., 2019; Henninger, 2021; Wang and Wu, 2011), for the following reasons. First, although these quantitative approaches enable an examination of unique respondents’ ICU profiles in terms of their type and intensity, the approaches require post hoc additional analyses, such as principal component analysis, to identify dominant response style factors. Therefore, these approaches are more suitable for measuring and controlling for ICU effects, with the aim of improving the precision of substantive trait estimates. Second, no available evidence has suggested that these approaches are effective under the data conditions used in the present study, mainly due to the large number of additional dimensions required by a response format that comprises many response categories. Therefore, we considered the mixture distribution IRT model to be the optimal approach to address our research question, given its focus on the typological nature of category usage as response behavior. Specifically, we expected that the mixture distribution IRT model would provide insight into how the proportion of RSs and the number of ordinarily used response categories change due to different lengths of rating scales in the self-chosen and given conditions. It should be noted that the term “ordinary response style”, which we use in the following, refers to a response pattern that is closest to the theoretical expectation, rather than to the absence of response tendencies. Therefore, the ordinary response pattern may deviate from the ideal expectation of equal distances between thresholds.

This study offers substantial extensions to previous research: First, it is the first study which examined the effects of ICU resulting from self-chosen and given rating scales on the psychometric quality of the data. Unlike prior studies involving self-defined rating scales, the process of selecting one of the offered rating scales should not be cognitively burdensome and time-intensive, as is typically the case with self-defined rating scales. Second, we worked systematically by first randomly assigning three rating scales to participants. This enabled us to investigate the effect of the number of response categories on the prevalence of response styles in the given condition. Subsequently, we allowed the same participants to choose between the three rating scales used in the previous step. This facilitated a comparison of scale usage among individuals who chose different rating scales. Third, we included personality traits to assess the criterion validity. Thus, the present study provides a comprehensive investigation of the psychometric quality of data obtained from self-chosen and given rating scales. The results of this study can inform researchers about the benefits of self-chosen rating scales in addressing ICU.

### Research questions and hypotheses

The present paper aims to analyze two general research questions.

First, we aim to investigate whether self-chosen rating scales and given rating scales differ in the types and sizes of response styles they produce. Therefore, we generated the following hypothesis:

- H1: The proportion of individuals who use the rating scale in an ordinary manner is larger in the self-chosen condition than in the given condition.

Second, we aim to examine the potential impact of different types of rating scales (administered by researchers or chosen by individuals) on the correlations between trait values and other variables of interest, such as personality traits. Therefore, we hypothesized the following:

- H2: The associations between individual trait value estimates of flourishing and external variables are influenced by the number of response categories in the given condition. In contrast, no differences in correlations arise when participants use self-chosen rating scales to respond to items. A plausible rationale for this discrepancy could be the prevalence of a larger extent of ICU in given rating scales in comparison to self-chosen rating scales, for which response format-related ICU is lower.

## Methods

### Data collection and sample

Participant recruitment and data collection occurred between February and July 2015 on Amazon’s Mechanical Turk (MTurk) platform. The MTurk platform is an online crowdsourcing labor market where MTurk workers complete various tasks, known as human intelligence tasks (HITs), for relatively low pay (Keith et al., 2017). This platform provides an online sample that is more representative of the general population in terms of psychological traits than student samples or samples recruited through online methods (McCredie & Morey, 2018). It also enables rapid, anonymous, and cost-effective gathering of high-quality data (Buhrmester et al., 2011; Mason & Suri, 2012). For this study, participants were required to meet the following inclusion criteria: being at least 18 years old, currently employed^1^, and residing in the United States. To minimize the risk of satisficing response behavior, only experienced MTurk workers who had competed a minimum of 100 approved HITs and maintained a high rate of positive feedback from requesters (with an approval rate of at least 95%) were eligible to participate (following the recommendations by Peer et al., 2014). The online questionnaire was generated using the SoSci Survey software package, a tool for conducting online surveys. To prevent duplicate participation, a filter was implemented based on the MTurk IDs of workers who had already taken part. Participants provided informed consent and received compensation of US$0.50 for their participation. The average response time was 16.35 minutes (*SD* = 5.32; *Md* = 15.67; *Q*_*1*_ = 12.70; *Q*_*3*_ = 19.27).

The entire sample comprised 7042 MTurk workers. Twenty participants did not respond to the flourishing items and were excluded from the analysis sample. As recommended by Curran (2016), 186 participants were excluded from the analysis dataset due to careless responses to ensure data quality. Careless clickers were defined as participants who demonstrated inattentive responding (incorrect responses to at least two of four instructional manipulation checks integrated into different sections of the online questionnaire),^2^ quick responding (response time quicker than the cutoff value, which was equal to the mean minus three standard deviations of the logarithmized response time variable),^3^ or invariant responses to at least half of the successive items of the cognitive tasks (verbal memory ability and verbal analogy tasks; for details on these measures, refer to the supplementary material, Part A). Thus, the analysis sample consisted of 6836 participants, 61.53% of whom were women. The average age of participants was 33.84 years (*SD* = 11.12). Almost all participants were native English speakers (97.18%). In terms of educational background, 8.68% of the participants reported having attained the lowest level of education (mostly high school completion), 27.09% had a college degree, 48.32% had a bachelor's or master's degree, and 3.60% had obtained a postgraduate degree.

### Study design

The study design consisted of three stages. In the first stage, a randomized between-subjects design was implemented, involving three given rating scales. The participants were randomly assigned to respond to the flourishing items with a four-, six-, or 11-point rating scale. We used the endpoint labeled rating scales, ranging from “strong disagree” to “strong agree”. The categories of the rating scales were provided with numerical values in ascending order, beginning at zero (e.g., for the four-point rating scale, ranging from 0 to 3). In the second stage, the participants were asked to respond to four sets of measures on sociodemographic variables, personality traits, cognitive tasks, and job-related variables (for details on the final two sets of measures, see supplementary material, Part A). These measures were used to check the quality of the randomization. No differences were detected between the randomized subsamples using univariate analysis of variance (ANOVA) for continuous variables and Pearson’s chi-square test for categorical variables (see Table S1 in the supplementary material for details). This outcome validated the effectiveness of the conducted randomization. In the third stage of the study, participants were presented with one flourishing item and three rating scales (as used in the first stage). They were instructed to choose the rating scale that they felt was most suitable for responding to this type of item. Next, the participants provided responses to the same flourishing items using the self-chosen rating scale. However, the choice of the rating scale was not influenced by the rating scale administered previously (refer to Table S2 in supplementary material). This was true for the entire sample (${\chi }^{2}$(4) = 6.67, *p* = .155) and for the analysis sample excluding careless clickers (${\chi }^{2}$(4) = 6.57, *p* = .161).

### Measures

#### The Flourishing Scale

(Diener et al., 2010) was conceptualized as a unidimensional scale and comprises eight items that assess an individual’s self-perceived social-psychological well-being, including positive functioning in important areas of life. The items describe aspects of human functioning ranging from competence, engagement, and interest to being an optimistic person, having supportive relationships, and leading a meaningful and purposeful life (e.g., ”I am engaged and interested in my daily activities.“ and “I am a good person and live a good life.”). The participants responded to the flourishing items using a four-, six-, or 11-point rating scale, depending on the subsample. In student samples, the flourishing scale has shown good psychometric properties (see, e.g., Diener et al., 2010; Ramirez-Maestre et al., 2017). In the current study, an exploratory factor analysis indicated that the flourishing scale had a single-factor structure in both study conditions and across all types of rating scales (first eigenvalue ranging from 4.68 to 5.34, variance explained by a single factor ranging from 58% to 67%, second eigenvalues ranging from 0.09 to 0.23, for more details, refer to Table S3 in the supplementary material). The scale has good internal consistency, with McDonald’s omegas ranging from .90 to .93.

#### Big Five

The short version of the Big Five Inventory (BFI-10; Rammstedt & John, 2007) was used to measure the five personality dimensions: extraversion, neuroticism, openness to experience, conscientiousness, and agreeableness. Each dimension comprises two prototypical items in the form of short phrases or adjectives (e.g., “is outgoing, sociable” for extraversion or “tends to be lazy” for conscientiousness). Participants rated the statements on a five-point rating scale, ranging from 1 (*disagree strongly*) to 5 (*agree strongly*). For each dimension, one of the items was negatively formulated and was recoded before the dimension scores were calculated. The BFI-10 has acceptable psychometric properties (Rammstedt & John, 2007). In the present study, the five-dimensional structure of this personality inventory exhibited an appropriate approximate fit when analyzed using CFA (${\chi }^{2}$(25) = 759.11, *p* < .001; RMSEA = .07, 90% CI [.06; .07]; CFI = .92; SRMR = .05; see Table S4 in the supplementary material for details). Regarding the short subscale length, subscale reliabilities were acceptable (McDonald’s $\omega$ = .71, .67, .51, .58, and .41 for extraversion, neuroticism, openness to experience, conscientiousness, and agreeableness subscales, respectively).

#### The Single Item Self-Esteem

(SISE; Robins et al., 2001) is a single-item measure of global self-esteem. The item “I have high self-esteem.” has acceptable psychometric properties and is a useful alternative to the Rosenberg self-esteem scale (RSE) for adult samples. Participants rated the item on a five-point scale, ranging from 1 (*not very true of me*) to 5 (*very true of me*).

#### The General Self-Efficacy Scale

(GSE; Schwarzer & Jerusalem, 1995) is a unidimensional self-report measure assessing individuals’ confidence in coping with challenging, stressful, or novel situations (e.g., “When I am confronted with a problem, I can usually find several solutions.” and “I can solve most problems if I invest the necessary effort.”). The GSE consists of ten items. Participants rated the items on a four-point rating scale with the following labels: 1 (*not at all true*), 2 (*hardly true*), 3 (*moderately true*), and 4 (*exactly true*). We validated the unidimensionality of the GSE scale using a one-factor CFA model, which indicated a good model fit (${\chi }^{2}$(35) = 293.94, *p* < .001; RMSEA = .03, 90% CI [.03; .04]; CFI = .99; SRMR = .04; see Table S5 in the supplementary material for details). Reliability (McDonald’s $\omega$) was .88.

#### Sociodemographic variables

Finally, the participants provided information about their age (in years), gender (1 = “female”, 2 = “male”), first language (1 = “English”, 2 = “other language”), educational level (1 = “high school graduate or less”, 2 = “some college,” 3 = “associate’s degree or bachelor’s degree”, 4 = “master’s degree, Ph.D., or professional degree”), and their job position (1 = “level 1: manager, self-employed, etc.”; 2 = “level 2: professional, researcher, etc.”; 3 = “level 3: technician, marketing, personal service worker, etc.”; 4 = “level 4: administrative worker, etc.”; 5 = “level 5: service worker, machine operator, MTurk worker, etc.”).

### Statistical analyses

#### Application of the mixed IRT model

To identify individual differences in ICU on the flourishing scale, we applied a restrictive version of the mixed generalized partial credit model (rmGPCM; for the GPCM, see Muraki, 1997; for the mGPCM, see von Davier & Yamamoto, 2007) to each subsample in both the self-chosen and given conditions. The rmGPCM assumes the existence of latent classes of individuals with homogeneous response patterns and thereby allows for variation in latent trait values within the latent classes. This model defines an individual’s probability of responding to a category of an item within a latent class as a logistic function of two item parameters: a class-specific threshold parameter (as the transition point between two adjacent categories) and a discrimination parameter that can vary across items but remains constant across latent classes (as a constraint). The equation for the rmGPCM is as follows:

1 $${P}_{vix}(\uptheta )={\sum }_{g=1}^{G}{\pi }_{g}\frac{exp [\sum_{s=0}^{x}{\delta }_{i}({\theta }_{vg}- {\tau }_{isg})]}{\sum_{c=0}^{m}exp[\sum_{s=0}^{c}{\delta }_{i}({\theta }_{vg}-{\tau }_{isg})]}$$

where ${P}_{vix}\left(\theta \right)$ denotes the probability of obtaining a response in category *x* (*x* ∈{0,..., *m*}) to categorical item *i* for an individual *v* assigned to latent class *g* with a latent trait value ${\theta }_{vg}$. Within a latent class *g*, the latent trait variable is assumed to be normally distributed, with a mean of zero and the freely estimated latent variance. A class-invariant discrimination parameter of item *i* is denoted by ${\delta }_{i}$ (with ${\delta }_{i}$ > 0 and with ${\updelta }_{1}$= 1), and a class-specific threshold parameter of item *i* is denoted by ${\tau }_{isg}$ (with *s* ∈{0,..., *c*} and ${\tau }_{isg}$= 0 for all *i* in all *g*). The sizes of the latent classes are considered model parameters (${\pi }_{g}$, with ${\sum }_{{\text{g}}=1}^{G}{\uppi }_{{\text{g}}}=$ 1). We estimated models with 1–5 classes and identified the best-fitting model within each subsample using the sample-size-adjusted Bayesian information criterion (saBIC; Sclove, 1987). This information criterion is appropriate for one-dimensional polytomous mixture distribution IRT models (see Kutscher et al., 2019; Sen & Cohen, 2023). The best-fitting model has the lowest saBIC value. Absolute model fit was assessed using bootstrap test statistics as Pearson and Cressie–Read χ2 goodness-of-fit statistics. A substantial subsample size (i.e., 2000–2500 individuals) is recommended for optimal model application (see, e.g., Cho, 2013; Huang, 2016; Jin & Wang, 2014; Kutscher et al., 2019). The rmGPCMs were specified and estimated using the Latent GOLD 6.0 software package (Vermunt & Magidson, 2021). The script is available in Part B of the supplementary material.

#### Interpretation of class-specific response styles

To interpret the class-specific response styles, we visualized the category characteristic curves (CCCs), illustrating the response probability of each item’s categories. The CCCs, which are based on the estimated item parameters of the best-fit model, indicate a segment of the latent continuum where a particular category has the highest probability of endorsement. Threshold parameters are located on the latent continuum and reflect the intersections of the CCCs of two adjacent categories. Ordered thresholds (${\tau }_{i, s-1}$ < ${\tau }_{i, s}$) indicate that each category of an item has a favored region on the latent continuum where its response probability is higher than that of other categories. Conversely, disordered thresholds indicate that individuals avoid a specific category (Andrich, 2010; Smith et al., 2011; Wetzel & Carstensen, 2014). Therefore, optimal response behavior implies ordered thresholds, and ordered thresholds, in turn, show that there is a segment on the latent continuum where the category is more likely to be selected than all other categories. Next, category widths, which represent the distances between adjacent thresholds, enable the identification of class-specific response styles. For example, in the case of an ERS, the extreme categories will have broader widths, while the intermediate categories will have narrower widths.

#### Comparison of ordinary response behavior

To test Hypothesis 1 regarding optimal response behavior, we created two binary variables to identify individuals belonging to the ORS classes in the self-chosen and given conditions. Within the randomized subsamples, we used the McNemar test for each group of individuals who chose a particular rating scale in the self-chosen condition. This test analyzed paired proportions and determine whether there were significant differences in optimal response behavior between the self-chosen and given conditions. A significant result in a one-tailed test would indicate a greater number of switchers from the non-ORS classes in the given condition to the ORS classes in the self-chosen condition (referred to as ${\widehat{\pi }}_{12}$) compared to the reverse switchers (referred to as ${\widehat{\pi }}_{21}$). The effect size was estimated using the population odds ratio (OR_pop_ = ${\widehat{\pi }}_{12}$/ ${\widehat{\pi }}_{21}$). To account for multiple comparisons, all proportion comparisons were performed at a corrected significance level (${\alpha }_{fam}$= 5%) using the Bonferroni correction approach. All analyses, unless otherwise reported, were performed in R version 4.3.0 (R Core Team, 2022).

#### Criterion validity

To test Hypothesis 2, we first examined the relationships between the individual trait value estimates of flourishing and the personality traits as external variables. To achieve this, we used Spearman’s rho correlation coefficient, because it is robust to violations of the bivariate normal distribution assumption. We chose this method because certain subsamples exhibited extreme values in the distribution of trait value estimates. We then conducted statistical tests to compare the correlation coefficients for each pair of randomized subsamples and examined differences in the correlations between the individual trait values of flourishing estimated under the given condition and the external variables. For this purpose, we followed the recommendation of Myers and Sirois (2004) by first converting the Spearman correlations into Pearson correlations and then transforming them to Fisher *z*-values. Bonferroni correction was applied to all conducted comparisons to adjust for multiple comparisons, which allowed us to examine whether the correlations varied depending on the number of response categories in the given rating scale.

We also used the same randomized subsamples from the given condition to compare the correlations between the external variables and the individual trait value estimates obtained in the self-chosen condition. Within each randomized subsample, three groups were created based on the rating scale chosen by individuals in the self-chosen condition: the group of individuals who chose the four-point rating scale, the group of individuals who chose the six-point rating scale, and the group of individuals who chose the 11-point rating scale. This enabled us to examine whether different correlations emerged when individuals responded to the items using a self-chosen rating scale, controlling for the randomized subsample from which they originated. A sensitivity analysis for the statistical test comparing two correlations from independent samples revealed that we can detect a minimum effect size of .19 using the significance test. The sensitivity analysis was conducted with a two-sided test, with the alpha level adjusted for multiple comparisons (1.7%), a power of 80%, and minimum sample sizes of *n*_*1*_ = 541 and *n*_*2*_ = 676 (corresponding to the groups from the subsample with the given 11-point rating scale). Because the Spearman’s rank correlation coefficient is a Pearson product-moment coefficient for ranks, the sensitivity analysis was conducted using GPower software version 3.1.9.7 (Faul et al., 2009) for the product-moment correlation (Statistics Solutions, n.d.).

#### Reliability

Reliability analyses were conducted for the manifest scores (including response styles), by calculating McDonald’s omega and the corresponding 95% confidence interval. Additionally, we reported a model-based reliability coefficient provided by the Latent GOLD 6.0 version and a bootstrapped 95% confidence interval for the best-fitting mixture model (Vermunt & Magidson, 2021). Latent GOLD estimates only one reliability coefficient (and not class-specific reliability coefficients) by regressing the latent variable on the observed items in a regression analysis. For this analysis, Latent GOLD 6.0 version defines the total variance as the sum of the variance of the class-specific latent trait scores and the average of the latent trait score variances (for more details, refer to Kim, 2012).

## Results

### Descriptive statistics for flourishing items

Table 1 presents the descriptive statistics for the flourishing items in both the self-chosen and the given conditions. Identical tendencies were observed across both conditions. The respondents rated aspects of their human functioning highly, including competence (item 5), quality of life (item 6), and optimism (item 7), while aspects such as supportive relationships (item 2) and engagement (item 3) were rated lower (except for the subsample that used a self-chosen four-point rating scale). The items showed a left-skewed distribution, indicating that the respondents were generally more satisfied than dissatisfied with their functioning in different aspects of their lives (for the plots depicting relative frequencies, see Fig. S1 in the supplementary material). The skewness became more pronounced as the number of response categories increased. For example, with a four-point rating scale, the bottom category represented less than 5% of the cases. However, the two upper categories were selected by a large proportion of the individuals (34–59%). For the six-point rating scale, the two lower categories had extremely low frequencies, while the top two categories were widely utilized (13–47%). Regarding the response format with 11 categories, the bottom five categories were sparsely used, but the upper four categories contained a high proportion of individuals (12–26%). The variability of responses also increased as the number of response categories increased.

**Table 1:** Descriptive statistics for the flourishing items under the self-chosen condition (top lines) and given condition (bottom lines)

	Four categories			Six categories			11 Categories		
Item	Mean (*SD*)	Skew.	Kurt.	Mean (*S*D)	Skew.	Kurt.	Mean (*SD*)	Skew.	Kurt.
Item 1. I lead a purposeful and meaningful life.	2.31 (0.80) 2.15 (0.77)	– 1.04 – 0.63	0.56 – 0.04	3.67 (1.01) 3.62 (1.15)	– 0.79 – 0.86	0.82 0.55	7.64 (2.10) 7.16 (2.23)	– 1.16 – 0.84	1.35 0.42
Item 2. My social relationships are supportive and rewarding.	2.17 (0.82) 2.10 (0.79)	– 0.69 – 0.52	– 0.23 – 0.32	3.49 (1.15) 3.60 (1.18)	– 0.77 – 0.84	0.24 0.34	7.24 (2.31) 7.01 (2.32)	– 0.99 – 0.82	0.61 0.19
Item 3. I am engaged and interested in my daily activities.	2.25 (0.77) 2.12 (0.77)	– 0.72 – 0.52	– 0.13 – 0.29	3.61 (1.06) 3.60 (1.12)	– 0.82 – 0.77	0.62 0.36	7.43 (2.14) 7.15 (2.21)	– 0.99 – 0.87	0.74 0.51
Item 4. I actively contribute to the happiness and well-being of others.	2.27 (0.76) 2.20 (0.74)	– 0.82 – 0.62	0.20 – 0.10	3.71 (1.04) 3.72 (1.11)	– 0.81 – 0.87	0.60 0.60	7.62 (2.15) 7.38 (2.17)	– 1.15 – 0.93	1.25 0.62
Item 5. I am competent and capable in the activities that are important to me.	2.53 (0.63) 2.49 (0.63)	– 1.17 – 0.97	1.22 0.44	4.09 (0.88) 4.13 (0.90)	– 1.13 – 1.21	1.77 2.14	8.25 (1.71) 8.10 (1.77)	– 1.35 – 1.19	2.39 1.70
Item 6. I am a good person and live a good life.	2.44 (0.68) 2.43 (0.67)	– 1.06 – 0.96	0.85 0.57	3.97 (0.95) 4.06 (0.92)	– 1.04 – 1.06	1.45 1.48	8.15 (1.80) 8.08 (1.81)	– 1.28 – 1.18	1.84 1.56
Item 7. I am optimistic about my future.	2.28 (0.81) 2.26 (0.80)	– 0.93 – 0.87	0.19 0.16	3.72 (1.15) 3.82 (1.16)	– 0.94 – 1.10	0.64 1.05	7.67 (2.36) 7.65 (2.29)	– 1.21 – 1.22	1.00 1.20
Item 8. People respect me.	2.18 (0.77) 2.20 (0.75)	– 0.68 – 0.68	0.01 0.13	3.65 (1.07) 3.77 (1.03)	– 0.90 – 0.95	0.80 1.21	7.51 (2.17) 7.50 (2.11)	– 1.11 – 1.07	1.07 1.05
McDonald’s omega ($\omega$), 95%-CI	.91 [.90; .94] .90 [.90; .90]			.90 [.89; .90] .91 [.91; .92]			.92 [.91; .93] .93 [.93; .94]		

*SD* = standard deviation; Skew. = skewness; Kurt. = kurtosis

The numerical value of the lowest category is always zero

### Best model solutions

Tables 2 and 3 show the goodness-of-fit statistics for the rmGPCMs with one to five latent classes in each subsample. In the self-chosen condition, the three-class model provided the best fit, indicating the lowest saBIC value across all rating scales (see Table 2). In the given condition, the three-class model was the best-fitting model in the subsample with a four-point rating scale, while the four-class models were best-fitting in the subsamples with six and 11 response categories (see Table 3). The absolute model fit statistics, which were computed for the best-class solutions, indicated a good fit of the model to the empirical data within each subsample (refer to the bootstrap Pearson and Cressie–Read χ2 test statistics in Tables 2 and 3). For all subsamples, the best-class models yielded interpretable class-specific patterns of category use. The mean assignment probabilities were also sufficiently high.

**Table 2:** Goodness-of-fit statistics for the rmGPCM in the self-chosen condition

Condition	Model	${N}_{par}$	LL	saBIC	Pearson *p* value (*SE*)	CR *p* value (*SE*)	Mean assignment probability in classes^1^	Estimated class size^1^	Model-based reliability estimates, 95% CI^2^
Four categories	1 class	32	– 10687	21510					
	2 classes	58	– 10393	21032					
	3 classes	84	– 10324	**21004**	.448 (0.02)	.604 (0.02)	.86, .81, .81	.58, .30, .13	.64 [.64; .65]
	4 classes	110	– 10283	21032					
	5 classes	136	– 10242	21060					
Six categories	1 class	48	– 28432	57097					
	2 classes	90	– 27532	55501					
	3 classes	132	– 27263	**55169**	.640 (0.02)	.152 (0.02)	.86, .77, .77	.58, .23, .19	.75 [.75; .75]
	4 classes	174	– 27173	55193					
	5 classes	216	– 27108	55267					
11 Categories	1 class	88	– 27282	54956					
	2 classes	170	– 26151	53058					
	3 classes	252	– 25738	**52598**	.902 (0.01)	.946 (0.01)	.92, .91, .83	.55, .25, .20	.82 [.82; .82]
	4 classes	334	– 25590	52667					
	5 classes	416	– 25504	52860					

${N}_{par}$ = number of model parameters; LL = log-likelihood; saBIC = sample-size adjusted Bayesian information criterion; Pearson *p* values (*SE*) = the bootstrapped *p* value and standard error corresponding to the Pearson ${\chi }^{2}$ goodness-of-fit statistics; CR *p* values (*SE*) = the bootstrapped *p* value and standard error corresponding to the Cressie–Read ${\chi }^{2}$ goodness-of-fit statistics

^1^ The values are reported in the following order: class with proper response style (ORS class), class with extreme response style (ERS class), and class with range response (RRS class)

^2^ The 95% confidence interval was calculated using the bootstrapped method implemented in Latent GOLD 6.0 version (Vermunt & Magidson, 2021)

Within each experimental condition, the BIC value of the best-fitting model is marked in bold

**Table 3:** Goodness-of-fit statistics for the rmGPCM in the given condition

Condition	Model	${N}_{par}$	LL	saBIC	Pearson *p* value (*SE*)	CR *p* value (*SE*)	Mean assignment probability in classes^1^	Estimated class size^1^	Model-based reliability estimates, 95% CI^2^
Four categories	1 class	32	– 14900	29946					
	2 classes	58	– 14535	29333					
	3 classes	84	– 14435	**29253**	.118 (0.01)	.504 (0.02)	.83, .84, .76	.46, .19, .35	.67 [.67; .67]
	4 classes	110	– 14381	29262					
	5 classes	136	– 14343	29304					
Six categories	1 class	48	– 20440	41099					
	2 classes	90	– 19544	39498					
	3 classes	132	– 19283	39168					
	4 classes	174	– 19182	**39158**	.822 (0.02)	.528 (0.02)	.84, .86, .76, .76	.42, .17, .18, .23	.69 [.69; .69]
	5 classes	216	– 19111	39208					
11 Categories	1 class	88	– 30317	61034					
	2 classes	170	– 28770	58313					
	3 classes	252	– 28322	57790					
	4 classes	334	– 28051	**57622**	.988 (0.00)	.990 (0.00)	.88, .86, .87, .88	.40, .14, .11, .34	.72 [.72; .72]
	5 classes	416	– 27963	57820					

${N}_{par}$ = number of model parameters; LL = log-likelihood; saBIC = sample-size adjusted Bayesian information criterion; Pearson *p* values (SE) = the bootstrapped *p* value and standard error corresponding to the Pearson ${\chi }^{2}$ goodness-of-fit statistics; CR *p* values (SE) = the bootstrapped *p* value and standard error corresponding to the Cressie–Read ${\chi }^{2}$ goodness-of-fit statistics

^1^ The values are reported in the following order: class with proper response style (ORS class), class with extreme response style (ERS class), class with range response (RRS class), and class with further response style

^2^ The 95% confidence interval was calculated using the bootstrapped method implemented in Latent GOLD 6.0 version (Vermunt & Magidson, 2021)

Within each experimental condition, the BIC value of the best-fitting model is marked in bold

### Scale usage

Table 4 summarizes the occurrence of disordered thresholds for the flourishing items in both conditions. First, the rate of disordered thresholds grew with increasing rating scale length, regardless of rating scale administration. For example, for the given rating scales, this affected up to 17, 48, and 63% of the thresholds when using four, six, and 11 response categories, respectively. This finding suggests that the respondents could differentiate between a few response categories but found it difficult to discriminate between finer gradations of a longer rating scale and to use the response format accurately. A similar trend was observed for the self-chosen rating scales, although fewer disordered thresholds were found in this condition. Respondents who chose the four-point rating scale also used it in an orderly manner, including all categories in the response process. For the self-chosen six-point rating scale, only one category was typically ignored, while for the 11-point rating scale, individuals avoided a maximum of five response categories. This suggests that the respondents understood and used their preferred rating scale better than the given rating scale. Second, in both conditions, the respondents were not homogeneous in terms of their category use but consistently showed class-specific patterns across different rating scales. In particular, the first classes in both the self-chosen and given conditions consistently used the response formats accurately, with disordered thresholds ranging from 0% to 24% across self-chosen rating scales and 0% to 9% across given response formats. It is worth noting that in the self-chosen condition, the participants condensed the 11-point rating scale into less meaningful categories compared to the given condition. Conversely, the second class showed a higher rate of category avoidance, accounting for 8–60% of disordered thresholds in the self-chosen condition and 17–63% in the given condition. Third, when a rating scale was chosen, there was less heterogeneity in category use, as evidenced by three latent classes in the self-chosen condition compared to four latent classes in the given condition (except for the four-point rating scale).

**Table 4:** Number and percentage of unordered thresholds for the flourishing items in the self-chosen and given conditions

	Self-chosen condition			Given condition		
	Four categories^1^	Six categories^1^	11 Categories^1^	Four categories^1^	Six categories^1^	11 Categories^1^
Number of unordered thresholds per item						
Class 1^2^ Class 2 Class 3 Class 4	0 0^4^ 0	0 0–1^5^ 0–1	1–3^6^ 4–5^7^ 2–3	0 0–1 0	0 1–3 0 0–1	0–1 5–8 1–2 1–4
Percentage of unordered thresholds in the scale^3^						
Class 1^2^ Class 2 Class 3 Class 4	0 8.33 0	0 22.50 15.00	23.75 60.00 25.00	0 16.67 0	0 47.50 0 12.50	8.75 62.50 18.75 26.25

*SD* = standard deviation

^1^ An item with four, six, and 11 response categories has 3, 5, and 10 thresholds, respectively

^2^ Latent classes are listed in the following order: class 1 with a proper response style (ORS class), class 2 with an extreme response style (ERS class), class 3 with a range response (RRS class), and class 4 with a further response style

^3^ The percentage of disordered thresholds in the scale was computed by summing the number of disordered thresholds across all items and then dividing it by the total sum of thresholds for all items

^4^ Out of the total eight items, six of them had all threshold parameters ordered, whereas only two items had a single unordered threshold

^5^ For six out of the eight items, either all threshold parameters were ordered, or only one threshold parameter was unordered, except for two remaining items which had two unordered thresholds

^6^ For seven out of the eight items, the number of unordered threshold parameters varied between one and three. The single remaining item had four unordered thresholds

^7^ Out of the total eight items, six of them had four or five unordered threshold parameters, while the remaining two items have seven and eight unordered thresholds, respectively

In the following section, we provide a comprehensive description of the class-specific differences in category use between the self-chosen and given conditions. To illustrate these differences, we have chosen two representative items from the flourishing scale: item 2 “My social relationships are supportive and rewarding.” and item 5 “I am competent and capable in the activities that are important to me.”

### Class-specific category use in the self-chosen condition

**Fig. 1:**
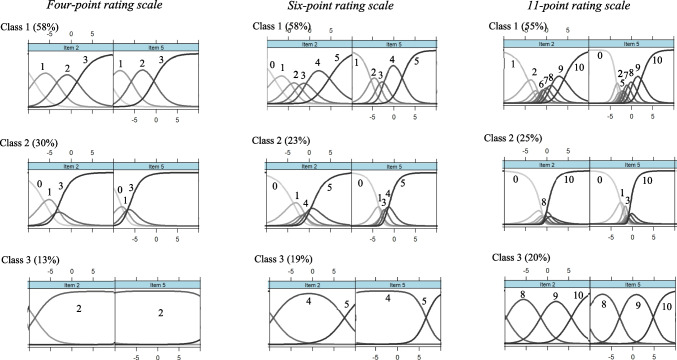
Class-specific category characteristic curves for the flourishing items in the self-chosen condition (values represent the categories with the highest response probability on a particular latent segment)

Figure 1 depicts the class-specific category characteristic curves (CCCs) for the two chosen items, which were administered using three rating scales in the self-chosen condition (refer to Figure S2 for all items; estimated item parameters are available in Tables S13–S15 in the supplementary material). For the four-point rating scale, the majority of the sample (58%) belonged to the first class, in which all four response categories covered equidistant segments on the latent continuum (refer to the first column in Fig. 1). In this class, the first category was located at the lower end of the latent continuum, indicating a low level of dissatisfaction with functioning in the life domains for very few individuals. The remaining sample was divided into two latent classes. The second class (30%), which was of intermediate size, exhibited a preference for the first and final response categories, which marked large areas of the latent continuum, while the intermediate categories were either avoided or marked narrow latent segments. In addition, the response categories in the second class were located closer to the lower end of the latent continuum, with the top category encompassing a larger portion of the reasonable range of values. In the third class (13%), which was the smallest one, only the penultimate category (*x* = 2) fell within the reasonable range of the latent continuum. Based on these distinctive patterns, we conclude that these three classes represent the ordinary response style (ORS class), the extreme response style (ERS class), and the range response style (RRS class), respectively.

Similar patterns of category use were observed for the six-point rating scale regarding the ORS class (58%), the ERS class (23%), and the RRS class (19%; see the middle column of Fig. 1). The large class effectively distinguished between five or six response categories, with the first category often avoided. The intermediate categories (*x* = 2 and *x* = 3) covered small segments of the latent continuum, while the middle class showed a preference for extreme categories, although some intermediate categories marked narrow latent segments (e.g., *x* = 1, or *x* = 3, or *x* = 4). The smallest class discriminated between two upper categories within a reasonable range of values (x = 4 and x = 5).

For the 11-point rating scale (see the third column in Fig. 1), the ORS class (55%) represented two to three lower (up to x = 3), one to two middle (x = 5, x = 6, or x = 7), and three upper response categories (x = 8 and above), depending on the item. The CCCs of the middle categories were close to each other, indicating that these categories were either avoided or marked very small latent segments. On average, individuals in this class discriminated between seven response categories. The ERS class (25%) predominantly used the first and final response categories, avoiding most categories in between. In the RRS class (20%), three broad upper categories were represented within the reasonable range of the latent continuum.

In summary, we found a consistent class structure across all three rating scales in the self-chosen condition. Three latent classes emerged with prototypical patterns of category use that consistently represented proportions of the sample: the ORS class at 55–58%, the ERS class at 24–29%, and the RRS class at 14–19%. The ORS class demonstrated optimal category use, effectively discriminating between all response categories for the four- and six-point rating scales. However, when using the 11-point rating scale, the ORS class showed less optimal category use, as the individuals could appropriately discriminate between only seven out of the 11 response categories. However, this level of discrimination can still be considered good compared to the other two classes. The ERS class exhibited a strong preference for extreme response categories, while also discriminating between some non-extreme categories when using the four- and six-point rating scales. The RRS class primarily used the second highest category for the four-point rating scale or two to three top categories for the six-point and 11-point rating scales. This suggests that, regardless of the rating scale chosen, individuals tend to focus on specific areas of the response format, such as the entire response format, omitting redundant categories, extreme categories, or specific top response categories.

### Class-specific category use in the given condition

**Fig. 2:**
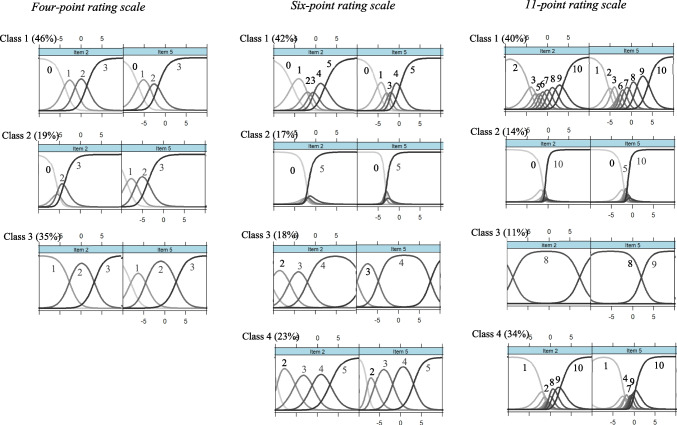
Class-specific category characteristic curves for the flourishing items in the given condition (values represent the categories with the highest response probability on a particular latent segment)

We observed several similarities in class-specific category use in the given condition (refer to Fig. 2; for the CCCs of all items, see Fig. S3, and for the estimated item parameters, refer to Tables S16–S18 in the supplementary material). For the four-point rating scale (left column in Fig. 2), optimal category use was observed in the first class (the ORS class; 46%). All thresholds were ordered, indicating that no response categories were avoided. The CCCs also demonstrated that the distances between the adjacent thresholds were approximately equidistant, indicating nearly equal widths of categories in this class. The second class (the ERS class; 19%) exhibited a preference for extreme categories that covered large latent segments, although one to two intermediate categories marked narrow latent segments. However, the CCCs were located toward the lower end of the latent continuum, so the top extreme category covered most of the meaningful range of values. In the third class (the RRS class; 35%), only the penultimate category (*x* = 2) was predominantly present within the reasonable latent range of values.

For the six-point rating scale (second column in Fig. 2), four latent classes with specific response patterns were identified. The first class (ORS class; 42%) distinguished between two lower categories and two upper categories, with the middle categories being either avoided or marking narrow latent areas. The second class (17%) exhibited a pure ERS, discriminating only between the two extreme categories. The third class (RRS class; 18%) had two broad categories (*x* = 3 and *x* = 4) within the meaningful range of the latent continuum. An additional class exhibited a specific type of range response style (the so-called extended range response style; eRRS class; 23%). Individuals in this class predominantly discriminated between the middle categories (*x* = 2 and *x* = 3) and the upper categories (*x* = 4 and *x* = 5).

Similar patterns were observed for the 11-point rating scale (third column in Fig. 2). The first class (ORS class; 40%) distinguished between one lower category (predominantly *x* = 2) and four upper categories (*x* = 7 and above), with one of the four intermediate categories being avoided and the other three intermediate categories marking narrow latent areas on the latent continuum. The second class (ERS class; 14%) showed a preference for extreme categories. In the third class (RRS class; 11%), the latent continuum was entirely covered by the third-to-last category (x = 8) and, for some items, by the penultimate category (*x* = 9). The fourth additional class (34%) discriminated between the two extreme categories, which covered a large portion of the latent continuum, and additionally between one to two lower categories and one to two upper categories, marking narrow sections. This class exhibited a type of ERS in which the individuals distinguished between multiple extreme categories on each pole of the response format. We referred to this class as the extended ERS class (eERS class).

In summary, a similar class structure, as observed with the self-chosen rating scales, was also evident for the given rating scales: the ORS class (40–46%), the ERS class (14–19%), and the RRS class (11–35%). The exception was the fourth classes for the six- and 11-point rating scales: the eRRS and eERS classes, respectively. The proportion of individuals with ORS increased as the number of response categories decreased, while the ERS class was present for each rating scale. However, for the four-point rating scale, the ERS class differed from the ERS class found for rating scales with six and 11 response categories by also differentiating between non-extreme response categories. In the RRS class, the penultimate upper category was predominantly preferred. When comparing ICU in the self-chosen and given conditions, the results suggested a higher occurrence of ICU in the given condition.

### Expected frequency distributions

**Fig. 3:**
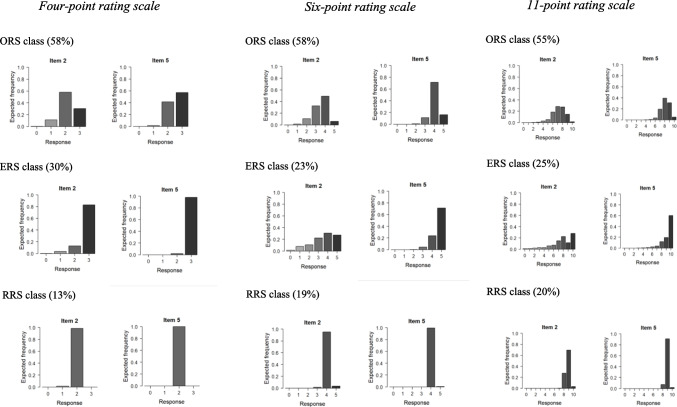
Expected relative frequencies for the flourishing items in the self-chosen condition

**Fig. 4:**
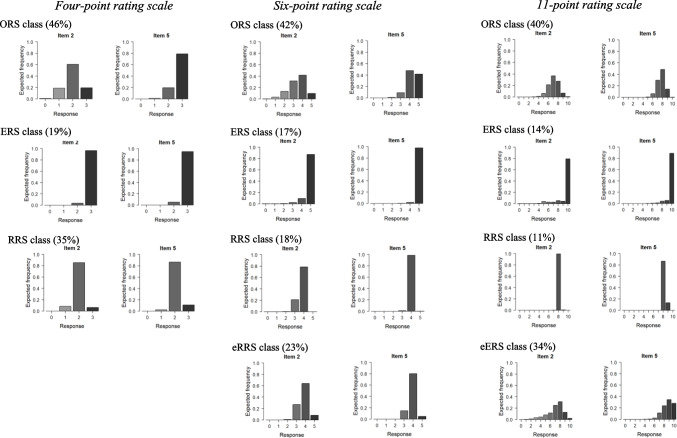
Expected relative frequencies for the flourishing items in the given condition

Figures 3 and 4 depict the expected relative frequency distributions for two selected flourishing items in the self-chosen and given conditions, respectively (for all items, refer to Figures S4–S5 in the supplementary material). Distributions are influenced by the class-specific threshold parameters and the latent trait distributions. Several similarities were observed for both conditions. First, regardless of the rating scale and type of administration, the lower categories were generally sparsely populated. Specifically, the four-point rating scale exhibited sparse data points at *x* = 0, the six-point rating scale showed sparsity from *x* = 0 to *x* = 2, and the 11-point rating scale displayed sparsity from *x* = 0 to *x* = 4. This suggests that individuals required fewer categories to express their dissatisfaction with functioning in different life domains. Second, the ORS class was expected to exhibit a preference for multiple top categories. Specifically, the four-point rating scale was expected to have preferences ranging from *x* = 2 to *x =* 3, the six-point rating scale from *x* = 3 to *x* = 5, and the 11-point rating scale from *x* = 6 to *x* = 9. The ERS class was expected to predominantly choose the top category, while the RRS class was expected to prefer the second-to-last category (except for the 11-point rating scale in the given condition, where the third-to-last category was expected to be preferred).

However, there were some differences in these expected distributions among the self-chosen and given conditions. Specifically, in the given condition, the eERS class exhibited stronger differentiation between response categories compared to the ERS and RRS classes. The distribution of the ERS class in the self-chosen condition displayed some similarity to the distribution of the eERS class in the given condition. This can be attributed to the fact that fewer latent classes appeared in the self-chosen condition.

### Ordinary response behavior in conditions

Table 5 presents the results from the comparison of the proportions of individuals using the ORS with the self-chosen and given rating scales. These results are given separately for the randomized subsamples. Consistently, a higher proportion of individuals were found to use the ORS with the self-chosen rating scales (54–61%) compared to the given rating scales (38–45%). In addition, the population odds ratio, which ranged from 1.71 to 3.38, indicated that the likelihood of individuals’ switching from a non-ORS style in the given rating scale to the ORS style in the self-chosen rating scale was approximately two to three times higher than the likelihood of individuals’ switching from the ORS style in the given rating scale to a non-ORS style in the self-chosen rating scale. Overall, these results confirmed Hypothesis 1 and strongly supported the effectiveness of the self-chosen rating scale in promoting the ORS when compared to the given rating scales.

**Table 5:** Comparison of proportion of individuals using the rating scale in an ordinary manner under the self-chosen and given conditions

Randomized Subsample	Group within randomized subsample that chose a particular rating scale	ORS (%) under self-chosen condition	ORS (%) under given condition	OR_pop_ = ${\widehat{\pi }}_{12}$ /${\widehat{\pi }}_{21}$	Test statistics
Given four categories	Chosen four categories	57.02	45.41	28.08 / 16.46 = 1.71	${\chi }^{2}$(1) = **17.47**, *p* < .001
	Chosen six categories	58.61	44.75	33.44 / 19.57 = 1.71	${\chi }^{2}$(1) = **35.57**, *p* < .001
	Chosen 11 categories	55.19	41.96	30.30 / 17.07 = 1.78	${\chi }^{2}$(1) = **25.97**, *p* < .001
Given six categories	Chosen four categories	56.75	38.14	30.47 / 11.86 = 2.57	${\chi }^{2}$(1) = **44.84**, *p* < .001
	Chosen six categories	60.80	43.10	32.25 / 14.55 = 2.22	${\chi }^{2}$(1) = **72.24**, *p* < .001
	Chosen 11 categories	54.06	41.51	29.25 / 16.69 = 1.75	${\chi }^{2}$(1) = **23.23**, *p* < .001
Given 11 categories	Chosen four categories	59.89	35.68	34.38 / 10.17 = 3.38	${\chi }^{2}$(1) = **71.21**, *p* < .001
	Chosen six categories	59.49	44.12	26.09 / 10.72 = 2.43	${\chi }^{2}$(1) = **67.64**, *p* < .001
	Chosen 11 categories	57.10	42.60	24.70 / 10.21 = 2.42	${\chi }^{2}$(1) = **40.69**, *p* < .001

ORS = ordinary response style

${\widehat{\pi }}_{12}$ = Proportion of switchers from non-ORS in the given condition to ORS in the self-chosen condition

${\widehat{\pi }}_{21}$ = Proportion of switchers form ORS in the given condition to non-ORS in the self-chosen condition

Within a randomized subsample, all comparisons were performed at a corrected significance level of .017 (${\alpha }_{fam}$ / 3) to test the specified directed hypothesis

### Correlations between flourishing trait values and external variables in conditions

Table 6 presents the Spearman correlations between personality traits and the trait values of flourishing estimated in the self-chosen and given conditions. In particular, when individuals used self-chosen rating scales, no statistically significant differences were found in the correlations between specific self-chosen rating scales within subsamples. This refers to the comparisons of the self-chosen four-point and six-point rating scales in the subsamples with given six and 11 response categories, as well as the comparisons of the self-chosen six-point and 11-point rating scales in the subsamples with given four and 11 response categories. However, there were three exceptions in which the correlations between two rating scales differed: (1) the self-chosen four-point scale versus the six-point scale for neuroticism, general self-efficacy, or self-esteem, depending on the randomized subsample; (2) the self-chosen four-point scale versus the 11-point scale for general self-efficacy (in the subsample with given four response categories); and (3) the six-point rating scale versus the 11-point rating scale for self-esteem (in the subsample with given 6 response categories). In contrast, when the given rating scales were used, the correlations with self-esteem (between the given four-point and both the six-point and 11-point rating scales), general self-efficacy (between the given four-point and both the six-point and 11-point rating scales), and neuroticism and openness to experience (between the given six-point and 11-point rating scales) were affected by the varying rating scales. These results partially supported Hypothesis 2, suggesting that the use of self-chosen rating scales partially eliminates the impact of the response format, specifically the number of response categories, on the observed correlations. In summary, these results highlighted two main insights. First, the number of response categories within a given rating scale can significantly affect the correlations. Second, the use of self-chosen rating scales offers notable benefits over the use of given rating scales.

**Table 6:** Comparisons of Spearman’s rho correlation coefficients between external variables and trait values of flourishing estimated under the self-chosen and given conditions

	Trait value estimates from the self-chosen condition									Trait value estimates from the given condition		
	Subsample with a given four-point rating scale			Subsample with a given six-point rating scale			Subsample with a given 11-point rating scale			Randomized subsamples		
	4 cat. (*n* = 577)	6 cat. (*n* = 981)	11 cat. (*n* = 703)	4 cat. (*n* = 548)	6 cat. (*n* = 1079)	11 cat. (*n* = 677)	4 cat. (*n* = 541)	6 cat. (*n* = 1054)	11 cat. (*n* = 676)	Given 4 categories (*n* = 2261)	Given 6 categories (*n* = 2304)	Given 11 categories (*n* = 2271)
Extraversion	.28	.27	.27	.26	.22	.28	.22	.23	.25	.28	.25	.25
Neuroticism	**– .25**^1^	**– .35**^1^	– .35	– .38	– .30	– .36	– .34	– .25	– .32	– .30	**– .32**^11^	**– .25**^11^
Openness to experience	.09*	.16	.10**	.09*	.11	.02^*ns*^	.05^*ns*^	.15	.07^*ns*^	.08	**.03**^*ns*12^	**.09**^12^
Conscientiousness	.34	.39	.37	.41	.33	.38	.36	.38	.36	.32	.31	.32
Agreeableness	.31	.31	.26	.27	.23	.25	.33	.29	.26	.27	.23	.26
Self-esteem	.49	.49	.47	**.53**^4^	**.43**^4,5^	**.52**^5^	**.56**^6^	**.43**^6^	.50	**.50**^7,9^	**.45**^7^	**.41**^9^
General self-efficacy	**.40**^2,3^	**.53**^2^	**.51**^3^	.41	.49	.48	.42	.47	.41	**.41**^8,10^	**.35**^8^	**.33**^10^

4 cat. = Group of individuals who chose a four-point rating scale to answer the flourishing items in the self-chosen condition. 6 cat. = Group of individuals who chose a six-point rating scale to answer the flourishing items in the self-chosen condition. 11 cat. = Group of individuals who chose an 11-point rating scale to answer the flourishing items in the self-chosen condition

All correlations are statistically significant at *p* < .001, with the exceptions noted in the table

^*ns*^ Non-significant correlation

Significant differences in correlations are indicated in bold.

Corrected significance level for all comparisons is .017 (${\alpha }_{fam}$ / 3)

^1^ *emp.z* = 2.16, *p* = .015

^2^ *emp.z* = – 3.33, *p* < .001

^3^ *emp.z* = – 2.63, *p* = .004

^4^ *emp.z* = 2.66, *p* = .004

^5^ *emp.z* = – 2.52, *p* = .006

^6^ *emp.z* = 3.20, *p* = .001

^7^ *emp.z* = 2.30, *p* = .011

^8^ *emp.z* = 2.80, *p* = .003

^9^ *emp.z* = 3.76, *p* < .001

^10^ *emp.z* = 3.30, *p* < .001

^11^ *emp.z* = – 2.72, *p* = .003

^12^ *emp.z* = – 2.13, *p* = .016

^*^ *p* < .05, ** *p* < .01

### Reliability

The flourishing scale showed excellent reliability for both the self-chosen and given rating scales, with McDonald’s *ω* coefficients greater than or equal to .90 (see Table 1). However, the reliability values for the corrected latent variables from the best-class models varied according to the number of response categories and the type of administration (see Tables 2 and 3). Reliability increased as the number of response categories increased. Specifically, the four-point rating scale had the lowest reliability (.64–.67), whereas the 11-point rating scale had the highest reliability (.72–.82). When comparing across conditions, minimal differences in reliability values were found for the four-point rating scale. However, the six- and 11-point rating scales were more reliable in the self-chosen condition.

The supplementary material (Part C) contains additional analyses that examine how individuals who chose different rating scales differed from each other in terms of sociodemographic, job-related variables, cognition-related indicators, and personality traits.

## Discussion

The main objective of this study was to compare the psychometric quality of data collected using self-chosen rating scales with the quality generated from given rating scales. The flourishing scale was used as an illustrative example. To conduct this comparison, the participants were first randomly assigned to three given rating scales with endpoint labeling: two shorter rating scales with four and six categories, respectively, and one longer rating scale with 11 categories. In the final stage of the survey, participants were instructed to choose one of the three rating scales and respond to the same flourishing items using their preferred self-chosen rating scale. To identify the response patterns elicited by the rating scales, the study employed the mixture distribution IRT approach, which enables exploration of response styles without ad hoc model specification. The occurrence of ICU served as the primary criterion to evaluate the construct validity, criterion validity, and reliability of the rating scales. The research questions and hypotheses aimed to investigate the benefits of self-chosen rating scales in addressing ICU and improving the measurement of psychological well-being.

### Occurrence of ICU and psychometric properties of self-chosen rating scales

The results confirmed our hypothesis that self-chosen rating scales allowed the respondents to provide more accurate responses compared to the given rating scales. First, for the self-chosen rating scales, respondents showed a consistent and less heterogeneous category use, regardless of the rating scale length. We found three classes with distinct responding: the ordinary response style (ORS; with occurrence ranging from 55% to 58%), extreme response style (ERS; ranging from 24% to 29%), and range response style (RRS; ranging from 14% to 19%). In contrast, the given rating scales showed more heterogeneous category use. Similar to the self-chosen condition, we identified the same three styles of responding – ORS (occurrence of 40–46%), ERS (of 14–19%), and RRS (of 11–35%) – as well as a fourth class. This fourth class had an extended range response style (eRRS; 23%) for the given six-point rating scale and an extended ERS (34%) with the given 11-point rating scale. Second, the proportion of respondents who used the rating scale in an ordinary manner was greater for self-chosen rating scales (54–61%) than for given rating scales (38–45%). In particular, for the self-chosen rating scales, the proportion of respondents with ORS remained nearly constant across different rating scale lengths, whereas for the given rating scales, ORS was more strongly associated with shorter rating scales. Third, the respondents with the self-chosen rating scales were better able to discriminate between response categories, resulting in fewer unordered response categories, compared to the given rating scales. In particular, the respondents with ORS effectively differentiated between all response categories on the four-point or six-point rating scale, and appropriately discriminated between seven response categories when using the 11-point rating scale, regardless of whether self-chosen or given rating scales were used. This suggests a better match between the respondents’ subjective response categories and their choice of rating scales, resulting in less cognitive burden and satisfying (Krosnick, 1991). Thus, this study has demonstrated that self-chosen rating scales are more effective in promoting accurate responses than given rating scales, leading to a positive effect on construct validity. However, self-chosen rating scales cannot completely eliminate trait-related ICU.

This study also provided evidence for the influence of the given rating scale on criterion validity, as demonstrated by correlations between latent trait value estimates of flourishing and external variables (e.g., neuroticism, openness to experience, self-esteem, and general self-efficacy), These correlations varied with rating scale length, indicating its influence. However, when the respondents used their preferred rating scale, the correlations were more consistent, regardless of the number of response options. These findings highlight two important points. First, the length of a given rating scale can significantly affect correlations due to ICU. This finding is consistent with that of previous research regarding the effects of ICU on the outcomes of statistical analyses (e.g., Khorramdel & von Davier, 2014; Plieninger, 2017; Tutz et al., 2018). Second, the use of self-chosen rating scales, which are less affected by response format-related ICU, offers a notable advantage over the use of a given rating scale. However, we were only able to partially confirm our second hypothesis, as we expected no differences in correlations for self-chosen rating scales. This might be due to the fact that we offered a limited number of rating scales from which to choose.

The study also examined the reliability of the flourishing scale using both self-chosen and given rating scales. McDonald’s omega demonstrated excellent reliability for the rating scales in both administration conditions. However, the model-based reliability of the corrected trait variables, after controlling for ICU effects, varied depending on rating scale length and administration condition. As indicated by previous research (e.g., Culpepper, 2013; Maydeu-Olivares et al., 2009), reliability tends to increase with the increased number of response categories. In this study, longer self-chosen rating scales (comprising six and 11 categories) demonstrated superior reliability, as they were less susceptible to trait-unrelated ICU. Furthermore, the self-chosen rating scales showed consistency in the assessment of the same construct, as evidenced by their factor structure, equivalent rank order of item means, and similar distribution shape. In brief, the findings of the present study highlighted the psychometric benefits of self-chosen rating scales in comparison to their traditionally used counterparts. This implies that enhancing the flexibility and customization of response formats can effectively address the challenges associated with given rating scales (e.g., response format-related ICU).

### Limitations and future research

One limitation of this study is related to its design, as the participants completed the flourishing items using both the given and self-chosen rating scales consecutively within a single survey. This introduced the potential for carryover effects, where the responses in the initial condition may have influenced the responses in the self-chosen condition. However, we demonstrated through statistical analyses that the choice of a rating scale was not influenced by the assignment of a rating scale. To address potential carryover effects, future studies could implement a washout period between the two administrations or utilize a more advanced four-group experimental design with two time points. The latter design would entail two groups alternating between the given and self-chosen conditions throughout the measurement time points, while the other two groups would consistently remain in their respective conditions for the duration of the study. This comprehensive design would enable researchers to assess the effects of both conditions and evaluate any possible carryover or order effects resulting from switching between conditions. Hence, a washout period or a four-group experimental design would improve the internal validity and interpretability of future studies that investigate similar research questions.

The present study utilized a typological modeling approach to examine the prevalence of ICU on the self-chosen and given rating scales. Future research could treat ICU types as continuous traits and apply models that allow for assessing the intensity of ICU in the data (for an overview, refer to Henninger & Meiser, 2020a, 2020b), or even models that allow for assessing the dynamics of ICU within a questionnaire (e.g., Merhof & Meiser, 2023). Such models would enable researchers to consider other criteria, such as variance estimates of ICU traits, when examining the effect of manipulating the rating scale administration on ICU. Furthermore, one reviewer noted the potential for confounding between high trait levels and a preference for extreme categories, particularly the highest category, when measuring substantial trait and response style effects using the same set of items in the presence of skewed response distributions (see also, Merhof et al., 2023). However, this so-called mimicry effect can impact most IRT models that assess response styles, including mixture IRT models, multidimensional nominal response models, and IRTree models with unidimensional pseudo-items. In this study, we expected longer rating scales to be less affected by this phenomenon because the respondents had more categories to distinguish between at the top of the response format. Future research could use innovative methods to estimate substantial traits and predefined response styles separately, for example, IRTree models with multidimensional pseudo-items proposed by Merhof et al. (2023).

The generalizability of this study’s results is limited by the specific construct, the utilized rating scales, and the study’s design. To validate and extend the findings, replication studies should be conducted using different constructs, rating scales with diverse features, and more sophisticated study designs. In this study, participants could choose from three endpoint-labeled rating scales, each with a varying number of response categories. Future research should also offer participants a broader range of rating scales to choose from, including those with fully labeled response categories that have been shown to reduce response bias and maximize variance (e.g., Eutsler & Lang, 2015). In addition, it would be interesting to examine participants’ preferences for specific features of rating scales and the variability of preferences within a given population. Although prior research has suggested that the number of response categories does not significantly impact certain measurement aspects, such as factor structure, measurement invariance, or latent mean differences (e.g., Xu & Leung, 2018), more research is required to ascertain whether rating scales with different features adequately capture the same underlying construct. One reviewer raised the interesting question of whether measurement invariance across the different subsamples who used different rating scales was necessary to compare the different groups. In this study, it was not possible to prove measurement invariance across the different rating scales used in the given and self-chosen conditions. This was due to the different measurement models based on the generalized partial credit model, which results from a different number of threshold parameters depending on the number of response categories in the rating scales. However, according to a meta-analysis analogy, the plausibility of the construct validity of diverse scales measuring the same construct lies in examining the correlation patterns of the construct of interest with external criterion variables. The observed similarity in correlation patterns for different rating scales in the present study confirmed the construct validity of the rating scales used in both conditions. Future research should broaden this focus beyond scrutinizing the construct of flourishing to validate the effectiveness of self-chosen rating scales in eliminating response format-related ICU or trait-unrelated ICU. Replication studies encompassing diverse and preferably multiple constructs are necessary to strengthen and generalize the findings, as ICU may include construct-specific components (e.g., Cabooter et al., 2017).

In general, further research should closely examine the response process of individuals who are assigned to a rating scale that does not align with their preferences in comparison to those using a rating scale that matches their preferences. This comparison can yield valuable insights into how individuals handle diverse rating scales. To support this investigation, we propose the use of multidimensional IRT models with random thresholds, particularly the random-effect generalized rating scale model (REGRSM; developed by Wang & Wu, 2011), or multidimensional IRT models for response styles that include additional dimensions to estimate individuals’ category preferences (e.g., Adams et al., 2019). These flexible models are applicable across various rating scales, providing researchers with insights into personalized usage of such scales and the intensity of response styles employed. Using these models can enhance the understanding of how individuals interact with different rating scales.

The results of the present study have significant implications for psychological assessment. First, by utilizing self-chosen rating scales, researchers and practitioners can enhance the accuracy and precision of psychological assessments, resulting in more robust findings. Specifically, the utilization of self-chosen rating scales effectively reduces or eliminates ICU related to response format, thereby improving the construct validity of the measurement and more accurately capturing the intended psychological construct. Additionally, data collected using self-chosen rating scales would be more reliable and would more accurately represent participants’ true trait or attitude, as systematic measurement errors associated with given rating scales are reduced. Second, offering participants a choice of rating scales enables researchers and practitioners to consider the diversity of preferences within the population, thus addressing the challenge of determining the most fitting rating scale for a particular population. Third, enabling participants to respond to items with their preferred rating scale boosts their engagement in the study and encourages them to provide more precise responses, leading to improved overall data quality.

## Conclusions

In conclusion, this study presents compelling evidence that self-chosen rating scales offer a valuable and efficient approach for obtaining high-quality data when measuring psychological constructs, as demonstrated by the flourishing scale employed in this research. Allowing individuals to choose their preferred rating scales can reduce ICU related to response format and consequently yield more accurate and reliable measurements. Although self-chosen rating scales cannot completely eliminate trait-related ICU, controlling for such effects leads to significantly improved psychometric quality compared to using given rating scales. Consequently, this study provides strong support for the effectiveness of self-chosen rating scales in enhancing data quality and emphasizes their relevance as a beneficial alternative to given rating scales in psychological assessment.

## Footnotes

1 The current study is a part of the project concerning the quality of rating scale for assessing job satisfaction, which is described in Kutscher and Eid (2020).

2 For example, “In order to verify that the program retains the data correctly, please select ‘hardly true’ for this statement.”

3 Another suggested cutoff is 2 s per item (Huang et al., 2012). Our cutoff was also consistent with this recommendation.

## Declarations

### Supplementary information

The online version contains supplementary material available at https://osf.io/xnuak/. This includes the results of additional analyses and the Latent GOLD code of the rmGPCM.
